# Method for Producing Perfect Joints in Gum-Section Plates

**Published:** 1906-07-15

**Authors:** R. E. Petty

**Affiliations:** Charleston, W. VA.


### A METHOD FOR PRODUCING PERFECT JOINTS IN GUM-SECTION PLATES.

BY R. E. PETTY, D.D.S., CHARLESTON, W. VA.

Of late years the tendency to discard the gum-section in artificial dentures seems to be so prevalent that a great many dentists do not use them at all, especially the younger men of the profession. This may be due to the fact that a more artistic assemblage can be secured with plain teeth, and, in many cases, a better articulation with less time. But with such a variety of good gum-section molds, how much more beautiful for "great exposure" are they than the dirty brown, once pink, vulcanite.

The following is a method by which the joints can be made invisible while in the mouth:

This method begins when the case is invested in the first half of the flask, and the plaster investment should only come to the wax border of the plate and in no case extending onto the wax.

Cut strips of tin foil No. 40, for each joint, making them one-fourth of an inch wide and one and a half inches long, and lay them over the joints, beginning on the labial surface of the front joint one-fourth of an inch below the porcelain and lapping the end over the cutting edge of the incisors onto the palatine surface of the wax. Burnish the foil well on to the teeth and over the joints. After each joint is so protected, pour the opposite side of the flask.

Heat the flask in boiling water for three minutes and separate. Remove the wax which will expose both ends of all the foil strips. Mix a thin cement and place on lingual surface of each joint, press the ends of the foil over the cement and joint.

Pack and vulcanize as usual, and on the removal of case from the flask, thoroughly wash and dry. Then remove the tin foil and the joints will be just as clear from coloring matter as they were when assembled on the articulator.

Dry the joints and slightly warm them, and with the wax spatula drop some hot wax into and over each joint. This will better protect them from dirt while finishing than the foil.

I have experimented with tin foil and cement for two years, using different thicknesses, and I find No. 40 foil best suited for the purpose. The entire method requires only a few minutes of time and the results will be very gratifying.

